# The use of an objective method (continuous exosomatic electrodermal activity without external stimuli) to evaluate patients with hyperhidrosis undergoing video-assisted sympathectomy

**DOI:** 10.31744/einstein_journal/2026AO1266

**Published:** 2026-04-07

**Authors:** Rafael José Silveira, Carolina Carvalho Jansen Sorbello, Nelson Wolosker, José Ribas Milanez de Campos, João José de Deus Cardoso, Alexandre Sherlley Casimiro Onofre

**Affiliations:** 1 Universidade Federal de Santa Catarina Hospital Universitário Department of Clinical Surgery Florianópolis SC Brazil Department of Clinical Surgery, Hospital Universitário, Universidade Federal de Santa Catarina, Florianópolis, SC, Brazil.; 2 Hospital Israelita Albert Einstein São Paulo SP Brazil Hospital Israelita Albert Einstein, São Paulo, SP, Brazil.; 3 Universidade de São Paulo Faculdade de Medicina Department of Vascular and Endovascular Surgery São Paulo SP Brazil Department of Vascular and Endovascular Surgery, Faculdade de Medicina, Universidade de São Paulo, São Paulo, SP, Brazil.; 4 Universidade de São Paulo Faculdade de Medicina Department of Thoracic Surgery São Paulo SP Brazil Department of Thoracic Surgery, Faculdade de Medicina, Universidade de São Paulo, São Paulo, SP, Brazil.; 5 Universidade Federal de Santa Catarina Hospital Universitário Department of Clinical Surgery Florianópolis SC Brazil Department of Clinical Surgery, Hospital Universitário, Universidade Federal de Santa Catarina, Florianópolis, SC, Brazil.; 6 Universidade Federal de Santa Catarina Department of Clinical Analysis Florianópolis SC Brazil Department of Clinical Analysis, Universidade Federal de Santa Catarina, Florianópolis, SC, Brazil.

**Keywords:** Hyperhidrosis, Sympathectomy, Electrodermal response, Excessive sweating, Surveys and questionnaires

## Abstract

**Objective:**

To analyze continuous exosomatic electrodermal activity (EDA) without external stimuli (EDAcw) in patients with primary hyperhidrosis before and after sympathectomy.

**Methods:**

This prospective study included 28 participants, categorized into two groups. The first group comprised 18 patients with palmoplantar hyperhidrosis who underwent bilateral thoracic sympathectomy. The second group (control) consisted of 10 patients. Two questionnaires were administered: the Control Group completed them once, whereas the Sympathectomy Group completed them preoperatively, and on the first and thirtieth postoperative days. Sweating was evaluated by measuring EDAcw using an MP36R biosensor. Measurements were obtained from the hands and feet for 5 min without external stimuli in an air-conditioned environment and were performed preoperatively, and on the first and thirtieth postoperative days. Anthropometric, clinical, and surgical data were collected, and no significant sociodemographic differences were observed between the groups.

**Results:**

In the Sympathectomy Group, quality of life improved, and sweating reduced. During preoperative assessment, EDA was higher in the hands and feet of patients with hyperhidrosis than in the Control Group. Postoperatively, EDA in the hands decreased, with 100% of the patients showing a decrease in sweating. For the feet, 67% of the patients reported reduced sweating, and 44% showed a statistically significant decline in EDA.

**Conclusion:**

Continuous exosomatic EDA measurement without external stimuli is a suitable method for assessing patients with palmoplantar hyperhidrosis and shows appropriate clinical correlation.

## In Brief

Continuous exosomatic electrodermal activity without external stimuli (EDAcw) was used to objectively assess patients with primary hyperhidrosis before and after sympathectomy. EDAcw correlated with clinical improvement and quality of life, confirming its reliability as a diagnostic and follow-up tool.

## Highlights

■ EDAcw objectively quantifies sweating in patients with hyperhidrosis.
■ Sympathectomy significantly reduced EDAcw and improved the quality of life.
■ The hands showed a 97% reduction in electrodermal activity postoperatively.
■ EDAcw is a reliable non-invasive tool for clinical evaluation and follow-up.

## Graphical abstract

**Figure:**
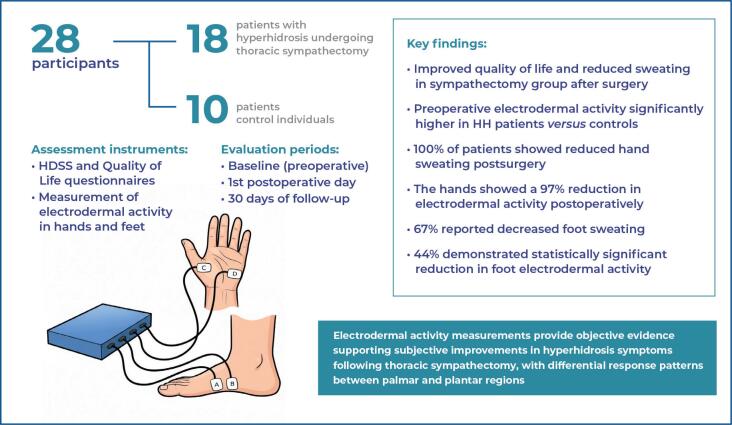

## INTRODUCTION

Primary hyperhidrosis is characterized by excessive sweating that is unrelated to external triggers or body temperature regulation.^(1)^ It is often associated with dysfunction of the sympathetic nervous system.^(2)^ This condition most commonly affects the palms of the hands, soles of the feet, and axilla, but can also be present on the face, trunk, and other parts of the body. Increased sweating can lead to embarrassment and negatively impact a person's social and psychological well-being,^(3)^ causing distress and reducing quality of life (QoL). Diagnosis is primarily based on a clinical assessment using questionnaires to measure sweating and evaluate its impact on the patient's QoL. The only definitive treatment is surgery; however, symptoms can be managed with medications such as oxybutynin.^(4,5)^

Video-assisted thoracoscopic thoracic sympathectomy (VATS) is considered one of the best treatment options for localized hyperhidrosis, owing to its effectiveness and safety.^(6)^ The level of intervention in the sympathetic chain is the main factor that influenced the results.^(7)^

Therefore, it is essential to evaluate the degree of sweating and the QoL when investigating this disease.^(8)^ Patients are typically given simple, easy-to-understand questionnaires to assess the intensity of sweating in the main affected areas of the body using scales such as the Hyperhidrosis Disease Severity Scale (HDSS).^(9)^ These questionnaires also help relate sweat volume to the repercussions of the interviewees’ routine activities.^(10)^ Questionnaires are cost-effective and non-invasive; however, they rely on the patient's personal interpretation and cognitive abilities, which can introduce subjectivity.^(11,12)^

Objective methods for quantifying sweat already described in the literature are not commonly used in medical practice. Some of the well-known objective methods include transepidermal sweat dosage and pad gloves (which quantify sweat transferred to a specific type of glove).^(13)^ However, the variability and lack of specificity of measurements, complex techniques, short measurement times, and the cyclical nature of hyperhidrosis make it challenging to use these measurements in clinical practice.

The electrodermal activity (EDA) represents the electrical properties of the skin resulting from sympathetic activity and causes the release of sweat. This alters the salt concentration in the cells and creates an electrical potential.^(14,15)^ This tool has been extensively used in psychological and behavioral studies and also in evaluating stress responses.^(16)^ Two methods are used for measuring the EDA: exosomatic (involving the application of an external electric current) and endosomatic (without an external electric current).^(17)^ Both techniques have been used in the past to study patients with hyperhidrosis and have yielded different results.^(18)^ However, the continuous measurement of EDA using the exosomatic technique without an external stimulus (EDAcw) has not been used to study patients with hyperhidrosis undergoing sympathectomy.

## OBJECTIVE

This study aimed to prospectively analyze the use of the exosomatic technique without external stimuli, utilizing a portable device to continuously measure electrodermal activity in patients with palmoplantar hyperhidrosis before and after surgical treatment, and to compare the results with data from individuals without the disease. Additionally, this study correlated these findings with established clinical diagnostic methods such as the Hyperhidrosis Disease Severity Scale and quality of life.

## METHODS

A prospective study involving 28 participants was conducted between January 2023 and January 2024. This study measured the intensity of sweating in 18 patients with palmoplantar hyperhidrosis who underwent VATS and in 10 individuals without hyperhidrosis using EDAcw. The study was approved by the Human Research Ethics Committee of *Universidade Federal de Santa Catarina* (CAAE: 43287321.8.0000.0121; # 4.712.363), and all participants provided informed consent.

The participants were categorized into two groups: the Sympathectomy Group, which comprised 18 (64.3%) patients with palmoplantar hyperhidrosis who underwent bilateral sympathectomy, and the Control Group, which consisted of 10 (35.7%) patients without hyperhidrosis or surgical intervention. Both groups had similar demographic characteristics, with a majority of female patients (72.2% *versus* 80.0%) who were in their third decade of life (mean age 25 *versus* 22.5 years) and had a body mass index below 25Kg/m^2^ (23.1 *versus* 22.1Kg/m^2^).

Patients in the Sympathectomy Group underwent bilateral sympathectomy (right side followed by the left side) of the fourth and fifth costal arches (R4/R5). The procedure involves monopolar electrocautery without a direct approach to the thoracic ganglion. Surgery was performed under general anesthesia with orotracheal intubation using a double-lumen tube, and non-invasive cardiovascular monitoring was conducted. Incisions of 0.5-1.0cm were made in the fifth intercostal space in the anterior axillary line to pass the optical system, and in the third intercostal space in the middle axillary line to introduce electrocautery after local infiltration with ropivacaine. All procedures were performed by the same team throughout the study period using a standardized surgical technique.

All patients except one were discharged on the first postoperative day. The remaining patient was discharged on the second postoperative day owing to residual pneumothorax and the need for pleural drainage. Compensatory hyperhidrosis (CH) was mild to moderate in ten patients (55.6%), severe in one patient (5.5%), and absent in seven patients (38.9%).

The participants underwent clinical assessment, completed questionnaires, and had their EDAcw measured on different occasions. The Control Group underwent these assessments once, while the Sympathectomy Group underwent them on three occasions: preoperatively, and on the first and thirtieth postoperative days. All the assessments were performed by the same investigator.

To measure the intensity of palmar and plantar hyperhidrosis, we used the HDSS, which was graded numerically from 1 to 4, representing the lowest to highest intensity of sweating.

To assess QoL, we used the quality-of-life questionnaire.^(19)^ The patients completed the QoL assessments independently, without any influence from the doctor. Preoperative QoL was categorized into five satisfaction levels based on the total scores obtained from the questionnaire: very poor (>84), poor (69-84), good (52-68), very good (36-51), and excellent (20-35). The QoL assessment was repeated 30 days postoperatively, and the patients were classified into one of five different levels of satisfaction according to their scores: much worse (>84), slightly worse (69-84), unchanged (52-68), slightly better (36-51), and much better (20-35).

To assess the EDAcw, we used an MP36R biosensor (Biopac Systems, Inc., USA) pre- and postoperatively. EDAcw was measured using an exosomatic technique with a constant electrical flow of 0.5 V. Disposable electrodes were placed on the thenar and hypothenar regions of the hands and the middle third of the medial surface of the feet. Measurements were taken after 10 min of rest, with participants seated comfortably, sequentially on the right and left hands, followed by the right and left feet, for five uninterrupted minutes in each location, without any specific stimulus, in a quiet environment with a temperature of 21-23^o^C and air humidity of 60-65%. The captured electrophysiological signals were digitized and transmitted to a computer system via a USB cable for analysis using Acknowledge software. The Control Group underwent electrodermal measurements only once.

Electrodermal activity was estimated in two ways: mean skin conductance (MSC) and skin conductance area (SCA) in the hands and feet at three different time points: preoperatively, and on the first and thirtieth postoperative days.

First, we analyzed the responses to the QoL and HDSS questionnaires and measured the average EDAcw and area in the Control and Sympathectomy Groups during each period (preoperatively, and on the first and thirtieth postoperative days). Then, we examined EDAcw separately in patient subgroups: those with improved hand sweating; those with significant or slight improvement in foot sweating, those with maintained foot sweating, and those with worsened foot sweating postoperatively.

### Statistical analysis of data

We first conducted a statistical analysis of the clinical data, followed by an analysis of the questionnaire responses (HDSS and QoL), and finally an examination of the EDA data.

The Shapiro-Wilk test was used to check the normality of the data. Numerical variables are described using central tendencies and dispersion measures, whereas categorical variables are described using absolute and relative frequencies. Student's *t*-test was used to analyze independent samples, and the Mann-Whitney U test was used to analyze the differences between groups. The Wilcoxon and Friedman tests were used to compare two and three different moments, respectively. A p≤0.05 was considered statistically significant. All data was analyzed using the R programming language version 4.2.1.

## RESULTS

The answers to the questionnaires and the EDA measurements are shown in table 1.

**Table 1:** Questionnaire responses (QoL and HDSS) and electrodermal activity measurements in the Control and Sympathectomy Groups at each analyzed period (preoperatively, on the first and thirtieth postoperative day)

Variable		Groups				p value
		Control	Sympathectomy			
			Baseline	1^th^ PO	30^th^ PO	
Quality of life‡,£		-	87.5 (80.8-93.3)	-	22.5 (20.8-28.5)	-
HDSS‡,£						
Hands		1	4.0 (3.0-4.0)	-	1.0 (1.0-1.0)	<0.001
Feet		1	3.0 (3.0-4.0)	-	2.0 (1.0-2.3)	0.03
EDA (average)‡,≠						
	Hands	5.15 (2.0-2.8)	11.3 (6.6-13.3)	0.3 (0.2-0.5)	1.3 (0.4-3.9)	<0.001
	Feet	2.35 (1.6-1.9)	11.2 (5.9-12.4)	0.3 (0.2-0.5)	1.2 (0.4-3.7)	<0.01
EDA (area)‡,≠						
	Hands	186.3 (53.2-390.7)	388.4 (284.3-630.2)	5.6 (1.1-17.9)	96.1 (20.0-130.0)	<0.001
	Feet	24.95 (10.8-156)	352.1 (278.7-647.7)	6.2 (1.3-18.4)	92.9 (18.0-128.6)	0.02

‡ values expressed as median and interquartile range (P25-75);

£ Wilcoxon test;

≠ Friedman test.

HDSS: Hyperhidrosis Disease Severity Scale; PO: postoperative; EDA: electrodermal activity; QoL: quality of life.

### Clinical assessment

We observed that the intensity of sweating on the hands, as measured by the HDSS preoperatively, was highest in the Sympathectomy Group (4.0) and decreased to a minimum level postoperatively, a value similar to that of the Control Group [z=-3.804; p<0.001].

For the feet, the average HDSS was 3.0 preoperatively and improved to 2.0 [z=-3.007; p<0.01] postoperatively, even surpassing that of the Control Group.

Furthermore, the patient's QoL improved postoperatively. Patients reported very poor QoL preoperatively, and showed significant improvement after sympathectomy [z=-3.726; p<0.001].

### EDA analysis

#### EDA in the hands

**Figure 1:**
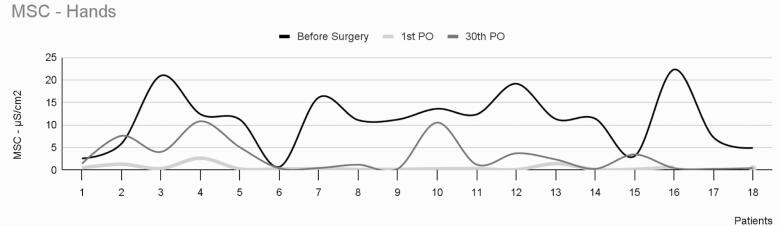
Mean skin conductance in the hands of 18 patients with palmar hyperhidrosis

The average intensity of EDAcw in the hands of the 18 patients who underwent surgery is shown in table 1 and figure 1. This study found statistically significant differences in EDAcw levels at three observation times: preoperatively, on the first and thirtieth day after sympathectomy. The comparative evaluation revealed differences between each time point: between the preoperative period and the first postoperative day (p<0.001), between the preoperative period and the thirtieth postoperative day (p=0.02), and between the first and thirtieth postoperative days (p<0.01)

MSC: mean skin conductance; 1st PO: first postoperative day; 30th PO: thirtieth postoperative day.

**Figure 2:**
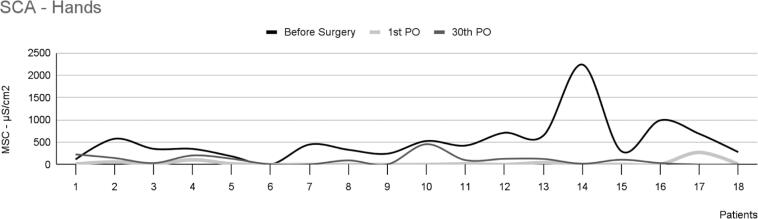
Skin conductance area in the hands of 18 patients with palmar hyperhidrosis

The intensity of EDAcw in the hands, as measured by area, is shown in table 1 and figure 2. A statistically significant difference was observed among the three observation times [χ^2^(2) 28.444; p<0.001]. When comparing each time point, significant differences were found between the preoperative period and the first postoperative day (p<0.001), between the preoperative period and the thirtieth postoperative day (p=0.01), and between the first and thirtieth postoperative days (p=0.01).

SCA: skin conductance area; 1st PO: first postoperative day; 30th PO: thirtieth postoperative day.

In the analyzed sample, only one patient (patient 12) experienced a slight decrease in sweating and showed a much smaller reduction in MSC between the preoperative period (13.6uS) and the thirtieth postoperative day (10.5uS), corresponding to a 23% reduction in the intensity of EDAcw 1 month postoperatively. The remaining 17 patients exhibited a decrease of approximately 97% in MSC over the same period. When we examined patient 12's sweating using SCA, we found no significant difference between the measurements taken preoperatively (527.7uS/cm^2^) and 30 days postoperatively (456.7uS/cm^2^). However, a noticeable decrease was observed in the EDA during the first postoperative day, indicating that the surgical procedure was performed correctly.

#### EDA in the feet

The average intensity of EDAcw in the feet of the 18 patients who had surgery showed a statistically significant difference between two of the three observation times [χ^2^(2) 10.941; p<0.01], which were between the preoperative period and the first postoperative day (p<0.01) and between the preoperative period and the thirtieth postoperative day (p=0.02). However, no significant difference was noted between the first and thirtieth postoperative days (p=0.61).

**Figure 3:**
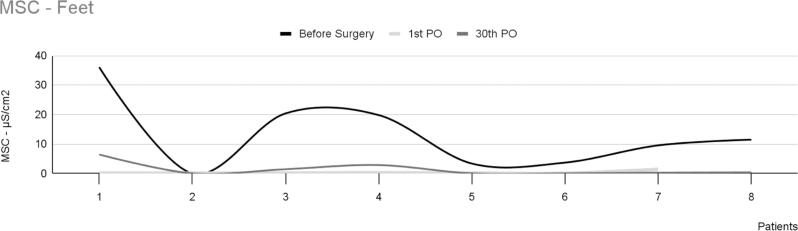
Mean skin conductance in the feet of eight patients with plantar hyperhidrosis, showing a significant decrease in sweating

**Figure 4:**
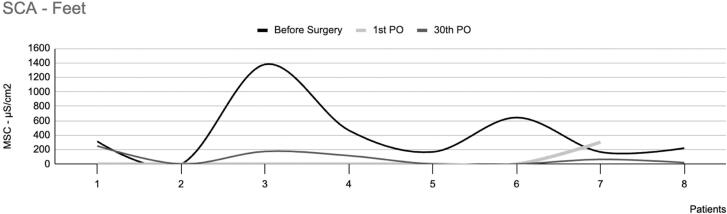
Skin conductance area in the feet of eight patients with plantar hyperhidrosis, showing a significant decrease in sweating

These patients have reported divergent results regarding reduced plantar sweating after sympathectomy. Of the 18 participants who underwent surgery, eight reported significantly reduced sweating. In these patients, the mean intensity of EDAcw showed a statistically significant difference between the preoperative period and the first postoperative day (p=0.05), and between the preoperative period and the thirtieth postoperative day (p=0.02). However, no significant difference was observed between the first and thirtieth postoperative days (p=0.60), as shown in figure 3. No statistically significant differences in EDAcw were observed in this group's area of the EDAcw (Figure 4).

MSC: mean skin conductance; 1st PO: first postoperative day; 30th PO: thirtieth postoperative day.

SCA: skin conductance area; 1st PO: first postoperative day; 30th PO: thirtieth postoperative day.

**Figure 5:**
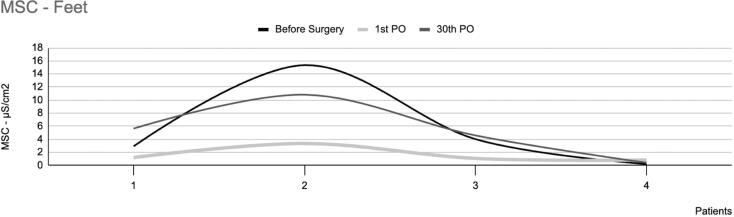
Mean skin conductance in the feet of four patients with plantar hyperhidrosis, showing a slight decrease in sweating

Of the remaining 10 patients, four reported a slight reduction in sweating. The mean EDAcw intensity and the EDAcw per area in this group showed no statistically significant differences across the three observation time points [χ^2^(2) 2.000; p=0.37], as shown in figure 5.

MSC: mean skin conductance; 1st PO: first postoperative day; 30th PO: thirtieth postoperative day.

**Figure 6:**
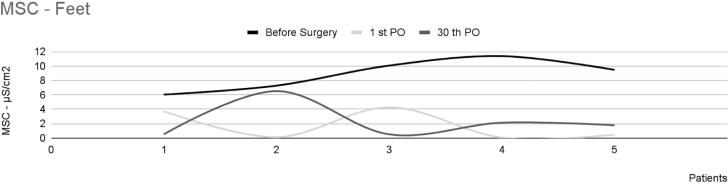
Mean skin conductance in the feet of five patients with plantar hyperhidrosis, showing no change in sweating

For the five patients who did not show any clinical change in plantar sweating, the intensity of EDAcw measured by MSC showed no statistically significant differences among the three observation time points [χ^2^(2) 5.200; p=0.07], as shown in figure 6.

MSC: mean skin conductance; 1st PO: first postoperative day; 30th PO: thirtieth postoperative day.

Only one patient reported an increase in postoperative plantar sweating; however, this finding was not clinically correlated with the EDAcw measurement, which decreased postoperatively.

## DISCUSSION

Hyperhidrosis is a disease with a significant psychosocial impact on patients’ lives, directly affecting their QoL and mental health. Patients who underwent sympathectomy showed considerable improvement in QoL.^(20)^ The results of this study confirmed the correlation between sympathectomy and a significant enhancement in patients’ QoL postoperatively.^(21)^

Decreased sweating is observed in approximately 70% of the patients undergoing medical treatment.^(22,23)^ However, surgical sympathectomy is the only definitive treatment for this disease, resulting in an improvement in 90% of palmoplantar hyperhidrosis cases.^(24)^ Since the 1990s, VATS has emerged as one of the best therapeutic options for localized hyperhidrosis.^(25)^ In our study, 97% of the patients showed significant improvements, with only one patient not achieving satisfactory results.

Improvement in patient QoL is the most important outcome of surgical treatments. This improvement is directly related to the control of sweating and the intensity of CH,^(26)^ with the level of intervention in the sympathetic chain being the most relevant technical aspect for achieving satisfactory results.

Thoracic sympathectomy is considered a safe procedure for treating hyperhidrosis with low morbidity and mortality rates. However, a complication that is often feared by patients is CH, which involves increased sweating in other areas of the body that were previously unaffected, such as the trunk and thighs. Several factors may contribute to CH, including the level of sympathetic denervation, the extent of sympathetic chain manipulation, and body mass index. Other, less common complications include pneumothorax, chylothorax, and hemorrhage.^(6)^

This study assessed VATS at the level of the fourth and fifth costal arches, and postoperative analysis showed a significant reduction in palmoplantar sweating in nearly all samples studied. In a review article, Nicolini et al. identified a trend toward intervention at this level, mainly for treating palmar hyperhidrosis, which has been reaffirmed in several other studies.^(27)^

In the context of plantar hyperhidrosis, VATS yields inconsistent results. In a retrospective multicenter study, Chen et al. reported a 29.3% improvement in plantar sweating after thoracic sympathectomy^(28)^ involving different surgical techniques. In another study, 40 patients underwent sympathectomies for hyperhidrosis. Approximately 45% of patients had decreased plantar sweating, 32% maintained stable sweating, and 22% experienced worsening conditions.^(29)^ This study indicated an overall symptom improvement in approximately 66% of patients. Although VATS does not specifically target plantar sweating, a significant percentage of patients may experience improvement.

In 2008, Tronstad et al. introduced a portable, non-invasive instrument capable of continuously measuring exosomatic EDA. This device is connected to a computer system and represents a significant advancement in medical research.^(30)^

Objective assessment of sweating was performed using the EDAcw, following the guidelines of the Society for Psychophysiological Research^(31)^ to prevent possible extrinsic and intrinsic factors that could bias the results. EDAcw levels were significantly higher in the hands and feet of the hyperhidrosis group compared to the group without hyperhidrosis, showing a strong clinical correlation. In a study by Manca et al.,^(32)^ Electrodermal activity was analyzed using the endosomatic technique in 10 participants with hyperhidrosis and 10 without hyperhidrosis, aged 26-50 years. They found that the hyperhidrosis group exhibited more sympathetic responses and shorter response latency time after sensory stimulation.

Electrodermal activity is an objective measure of sympathetic activity in eccrine sweat glands. Its electrophysiological origin is in the thermoregulatory center of the hypothalamus; however, it is influenced by the entire cerebral cortex, particularly the limbic system.^(33)^ Van Dooren et al.^(34)^ evaluated the EDA of 17 participants in 16 dermatomes simultaneously for 3 min at each site, showing the relationship between this measurement and the density of the sweat glands and their stimulation level. In a comparative study, Machado-Moreira et al.^(35)^ found that EDA levels increased earlier than sweat levels in the four dermatomes of 14 participants in response to increased body temperature, suggesting that this measurement precedes the presence of sweat.

Skin conductance is the key electrophysiological parameter analyzed using EDA. Skin conductance can be obtained using exosomatic or endosomatic techniques, depending on whether an electrical current needs to be applied. This measurement is classified into skin conductance level, which represents slow and gradual variations in skin conductance and is considered the baseline or "background" level of sympathetic activity, and skin conductance response or skin sympathetic response, which involves a sudden increase in the amplitude of skin conductance that can occur spontaneously (non-specific) or in response to a specific stimulus (specific).^(36)^

Regarding the effects of thoracic sympathectomy on EDA, Lefaucheur et al.^(37)^ reported a decrease in the amplitude of sympathetic responses in the hands on the side subjected to sympathectomy and on the non-operated side. This suggests a neuroplastic change at the central level of synaptic connections postoperatively. However, no significant changes were observed in the feet. In contrast, Lewis et al.^(38)^ found no significant differences in EDA after VATS in 26 patients with hyperhidrosis.

In this study, using EDAcw, we detected a significant reduction in the skin conductance measurements of the hands and feet. This reduction was more pronounced on the first postoperative day compared to the thirtieth postoperative day, suggesting a neuroplastic modification throughout the sympathetic chain after thoracic sympathectomy that changed over time. Another possible explanation for this difference is the postsurgical plateau effect. Since improvement in sweating after sympathectomy occurs rapidly, a significant quantitative difference between evaluations on the first and thirtieth postoperative days is not expected. Patients will be re-evaluated at 6 months and 1 year after sympathectomy to assess the long-term results.

EDAcw measurements have proven to be effective in assessing sympathetic activity in sweat glands, and consequently, the intensity of sweating, sensitively and accurately. Therefore, EDAcw can be considered a valuable tool for the objective evaluation of patients with hyperhidrosis. New instruments capable of continuously and efficiently measuring EDAcw in various clinical scenarios, while interfacing with a computer system, allow not only real-time assessment, but also data storage in the cloud. This enables review of the obtained measurements and the analysis of other electrophysiological parameters. These features can contribute to a better understanding of the pathophysiology of hyperhidrosis and facilitate the classification and determination of therapeutic approaches, particularly in relation to different thoracic sympathectomy techniques.

## CONCLUSION

A prospective analysis of EDAcw measurement using a portable device in individuals with palmoplantar hyperhidrosis, conducted preoperatively and postoperatively, and compared with data from individuals without the disease, proved to be a sensitive and efficient objective measure. It correlated well with established clinical diagnostic methods, showing statistically significant differences in EDAcw levels in the hands and feet between the control and hyperhidrosis groups, and preoperative and postoperative assessments within the hyperhidrosis group.

## Data Availability

The underlying content is contained within the manuscript.
